# Influenza A virus causes maternal and fetal pathology via innate and adaptive vascular inflammation in mice

**DOI:** 10.1073/pnas.2006905117

**Published:** 2020-09-21

**Authors:** Stella Liong, Osezua Oseghale, Eunice E. To, Kurt Brassington, Jonathan R. Erlich, Raymond Luong, Felicia Liong, Robert Brooks, Cara Martin, Sharon O’Toole, Antony Vinh, Luke A. J. O’Neill, Steven Bozinovski, Ross Vlahos, Paris C. Papagianis, John J. O’Leary, Doug A. Brooks, Stavros Selemidis

**Affiliations:** ^a^School of Health and Biomedical Sciences, Royal Melbourne Institute of Technology University, Bundoora, VIC 3083, Australia;; ^b^Department of Pharmacology, Biomedicine Discovery Institute, Monash University, Clayton, VIC 3800, Australia;; ^c^Clinical and Health Sciences, University of South Australia, Adelaide, SA 5001, Australia;; ^d^Discipline of Histopathology, School of Medicine, Trinity Translational Medicine Institute, Trinity College Dublin, Dublin 2, Ireland;; ^e^Sir Patrick Dun’s Laboratory, Central Pathology Laboratory, St James’s Hospital, Dublin 8, Ireland;; ^f^Emer Casey Research Laboratory, Molecular Pathology Laboratory, The Coombe Women and Infants University Hospital, Dublin 8, Ireland;; ^g^Irish Cervical Screening Research Consortium (CERVIVA), Trinity College Dublin, Dublin 2, Ireland;; ^h^Department of Physiology, Anatomy and Microbiology, School of Life Sciences, La Trobe University, Melbourne Campus, Bundoora, VIC 3086, Australia;; ^i^School of Biochemistry and Immunology, Trinity Biomedical Sciences Institute, Trinity College Dublin, Dublin 2, Ireland

**Keywords:** pregnancy, influenza, inflammation

## Abstract

Influenza A virus (IAV) infection during pregnancy causes severe maternal and perinatal complications, despite a lack of vertical transmission of IAV across the placenta. Here, we demonstrate a significant alteration in the maternal vascular landscape that underpins the maternal and downstream fetal pathology to IAV infection in mice. In IAV infection of nonpregnant mice, the local lung inflammatory response was contained to the lungs and was self-resolving, whereas in pregnant mice, virus dissemination to major maternal blood vessels, including the aorta, resulted in a peripheral "vascular storm," with elevated proinflammatory and antiviral mediators and the influx of Ly6C^low^ and Ly6C^high^ monocytes, plus neutrophils and T cells. This vascular storm was associated with elevated levels of the adhesion molecules ICAM and VCAM and the pattern-recognition receptors TLR7 and TLR9 in the vascular wall, resulting in profound vascular dysfunction. The sequalae of this IAV-driven vascular storm included placental growth retardation and intrauterine growth restriction, evidence of placental and fetal brain hypoxia, and increased circulating cell free fetal DNA and soluble Flt1. In contrast, IAV infection in nonpregnant mice caused no obvious alterations in endothelial function or vascular inflammation. Therefore, IAV infection during pregnancy drives a significant systemic vascular alteration in pregnant dams, which likely suppresses critical blood flow to the placenta and fetus. This study in mice provides a fundamental mechanistic insight and a paradigm into how an immune response to a respiratory virus, such as IAV, is likely to specifically drive maternal and fetal pathologies during pregnancy.

## Significance

Influenza infection during pregnancy is associated with increased maternal and perinatal complications. Here, we show that, during pregnancy, influenza infection leads to viral dissemination into the aorta, resulting in a peripheral “vascular storm” characterized by enhanced inflammatory mediators; the influx of Ly6C monocytes, neutrophils, and T cells; and impaired vascular function. The ensuing vascular storm induced hypoxia in the placenta and fetal brain and caused an increase in circulating cell free fetal DNA and soluble Flt1 release. We demonstrate that vascular dysfunction occurs in response to viral infection during pregnancy, which may explain the high rates of morbidity and mortality in pregnant dams, as well as the downstream perinatal complications associated with influenza infection.

Influenza A virus (IAV) infection during pregnancy is a very important public health concern, given that women will invariably encounter an influenza season during their term. Pregnant women are more likely to experience complications associated with IAV infection, such as acute cardiopulmonary events, pneumonia, and acute respiratory distress syndrome. Maternal IAV infection can also lead to neonatal complications, such as seizures, cerebral palsy, intrauterine growth restriction (IUGR), preterm birth, and neonatal death. Moreover, maternal IAV infection has been strongly associated with long-term health conditions in the offspring, including cardiovascular disease and schizophrenia (1). The mechanisms for increased maternal and fetal morbidity/mortality during IAV infection are currently unknown. Unlike Zika virus (2), vertical transmission of IAV to the placenta and fetus does not occur, and, therefore, IAV-induced pathology in the offspring cannot be attributed to a direct cytolytic effect of the virus per se. This property of IAV and the pathogenesis it causes during pregnancy remain poorly characterized.

Pregnancy has profound effects on the maternal cardiovascular system to support the developing fetus. Increased production of endothelial nitric oxide (NO) and other vasodilators lead to a reduction in systemic vascular resistance (25 to 30%), which is compensated for by increases in cardiac output (up to 40%) and stroke volume (3, 4). However, abnormal adaptations or vascular dysfunction during pregnancy can lead to very severe complications for both the mother and baby. One example is preeclampsia, a disorder characterized by hypertension and proteinuria, which can lead to multiorgan failure and even death. Preeclampsia is associated with chronic immune activation, resulting in endothelial inflammation and dysfunction (5, 6). This placental vascular disorder develops in part due to an angiogenic imbalance. Preclinical and clinical studies suggest that the significant increase in anti-angiogenic soluble fms-like tyrosine 1 (sFLT1) inhibits proangiogenic factors, such as vascular endothelial growth factor A (VEGF-A) signaling, consequently prompting vascular alterations and chronic placental under perfusion (7, 8). The reduced placental blood flow, resulting in IUGR and fetal hypoxia (9), has significant long-term implications in the adult. Importantly, women receiving an influenza vaccine during pregnancy have decreased rates of severe preeclampsia (10). Moreover, preeclampsia significantly increases the risk of future cardiovascular disease in women, with a fourfold to fivefold increased risk of developing hypertension (11).

In the present study, we have identified a hitherto-unappreciated respiratory, placental, and cardiovascular axis of pathology that IAV drives in dams, which results in complications in the fetus, including IUGR and fetal hypoxia. A central feature of this IAV pathogenesis is an exacerbated inflammatory response in the maternal host, particularly within large blood vessels, including the aorta, resulting in maternal vascular inflammation and endothelial dysfunction, contributing to placental and fetal hypoxia. In addition, IAV-infected pregnant dams are associated with placental stress, as measured by the release of highly inflammatory cell-free fetal DNA (CFFDNA) (12), as well as the secretion of the antiangiogenic protein sFLT1 from the placenta. Of relevance to this study, sFLT1 has been shown to sensitize endothelial cells to proinflammatory factors (13). Consequently, the release of CFFDNA and sFLT1 into the maternal circulation promotes an exacerbated systemic inflammatory response. Therefore, IAV infection during pregnancy presents a *quashing conundrum* for the maternal immune system, whereby the IAV and the growing fetus become a critical protagonist of a "vascular storm," which can ultimately bring upon adverse health or fatal outcomes for both the dam and offspring. Consequently, influenza in pregnancy should *not* be considered as an isolated acute event, but, rather, one that initiates a pathogenic downstream sequelae of events, which can have profound consequences in the long term for maternal and offspring health.

## Results

### IAV Infection in Pregnancy Drives an Exacerbated Systemic Inflammatory Response.

Here, we have used a mouse model of IAV infection in pregnancy that recapitulates pathological and immunological features of human IAV infection. We investigated the infection of pregnant mice at embryonic day 12 (E12) gestation (human equivalent of second trimester of pregnancy) with a moderately pathogenic strain of IAV (HKx31; 10^4^ plaque-forming units [PFUs]) at a dose that causes predominantly a local and resolving lung infection in nonpregnant mice, mimicking seasonal influenza symptoms. In nonpregnant mice, IAV infection resulted in a significant increase in airway inflammation at 3 d postinfection (dpi), which was characterized by infiltrating macrophages, neutrophils, and lymphocytes (*SI Appendix*, Fig. S1*A*). In the lung tissue, there was a significant elevation in the expression of the typical proinflammatory cytokines to IAV infection, including interleukin 6 (IL-6), tumor necrosis factor α (TNF-α), IL-1β, and interferon-γ (IFN-γ) (*SI Appendix*, Fig. S1*B*); chemokines CCL3 and CXCL2; colony-stimulating factor-3 (CSF-3) (*SI Appendix*, Fig. S1*C*), and antiviral mediator IFN-β (*SI Appendix*, Fig. S1*D*). IAV infection resulted in only a modest systemic inflammatory response, with small increases in circulating neutrophils and lymphocytes, but no alteration in circulating lymphocytes or platelets (*SI Appendix*, Fig. S1*E*). The current paradigm suggests that the maternal immune system is largely in a state of immunosuppression during pregnancy and that this can have a devastating impact on the growing fetus (14). However, it is believed that this immunosuppression of the antiviral immune response heightens the risk and severity of an infectious agent such as IAV. In contrast to this current consensus, we observed a modest, but significantly elevated, airway inflammatory response to IAV infection during pregnancy, compared to infected nonpregnant mice (*SI Appendix*, Fig. S1*A*). This enhanced airway inflammation was attributed to heightened macrophage, neutrophil, and lymphocyte infiltration within the airways of pregnant mice (*SI Appendix*, Fig. S1*A*). We show that proinflammatory cytokines to IAV infection, including IL-6, TNF-α, IL-1β, and IFN-γ (*SI Appendix*, Fig. S1*B*); chemokines CCL3 and CXCL2; CSF-3 (*SI Appendix*, Fig. S1*C*); and IFN-β (*SI Appendix*, Fig. S1*D*) were elevated in the lungs of pregnant mice, but these responses were not significantly different from those obtained in nonpregnant mice. However, IAV infection in pregnant mice resulted in significant elevation in systemic inflammation, with increases in circulating neutrophils, lymphocytes, and platelets (*SI Appendix*, Fig. S1*E*). Interestingly, IAV in pregnancy resulted in elevated numbers of systemic neutrophils, lymphocytes, and platelets compared to nonpregnant mice (*SI Appendix*, Fig. S1*E*). Overall, IAV infection in pregnancy resulted in a local lung inflammatory response that was modestly larger in magnitude to that observed in nonpregnant mice; however, there was a significantly larger systemic response, which suggests that influenza pathology in pregnancy is likely to extend beyond the lung to include peripheral consequences.

### Maternal IAV Infection Is Associated with Placental Growth Retardation and Hypoxia and Fetal Brain Hypoxia.

**Fig. 1.**
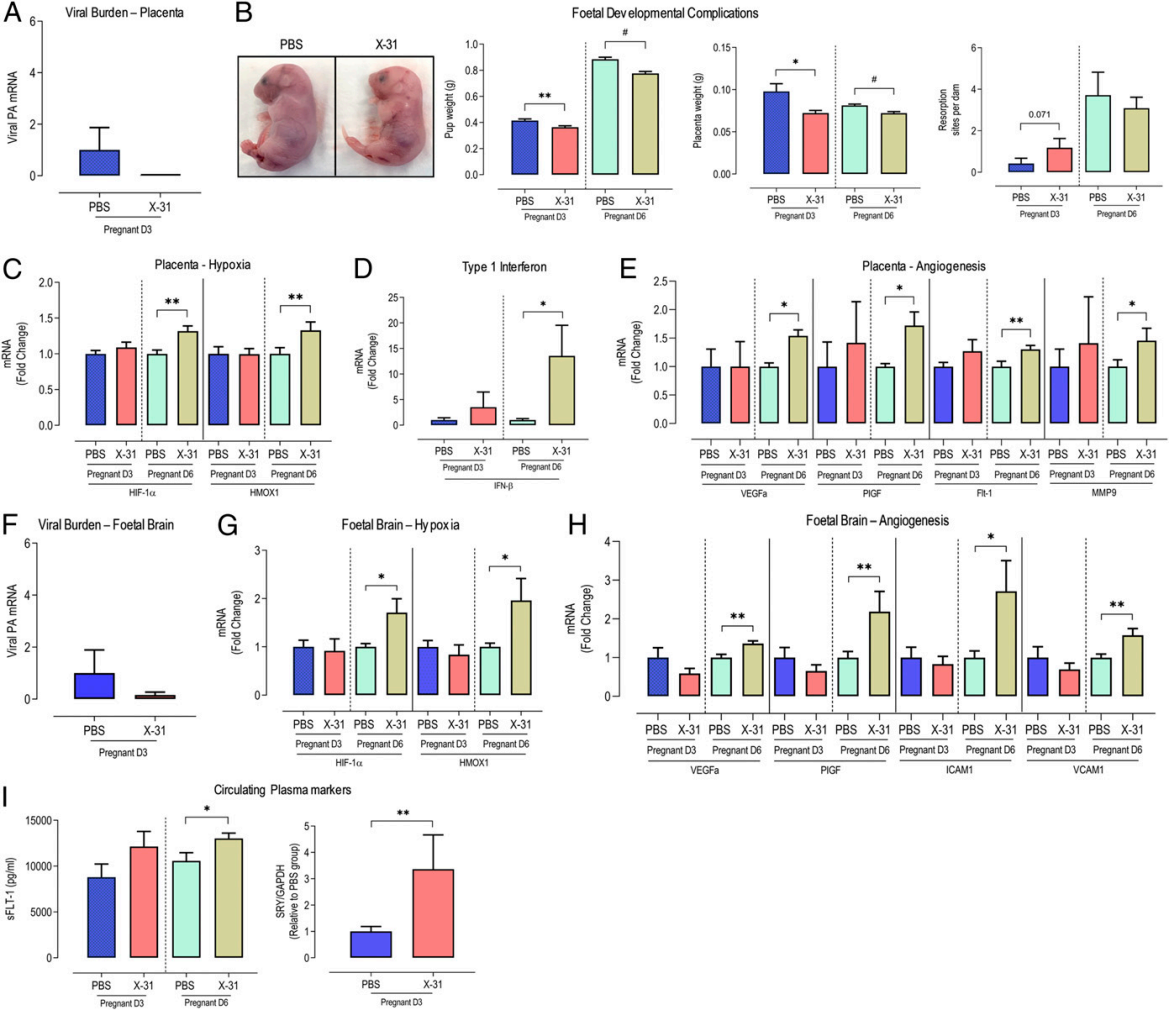
Seasonal IAV infection in pregnant mice is associated with placental and fetal brain hypoxia and angiogenesis. Eight- to 12-wk-old pregnant (E12 gestation) C57BL/6 mice were i.n. inoculated with PBS or Hk-x31 (X-31; 10^4^ PFU) for fetal assessment at 3 dpi (D3) and 6 dpi (D6). (*A*) IAV burden in placenta was quantified using qPCR by measuring the mRNA expression of the IAV segment 3 polymerase (PA). (*B*) Representative images showing pups from PBS- and X-31–infected dams. Pup weight (grams), placental weight, and number of resorption sites were recorded per dam. (*C*) Placental mRNA expression of hypoxic markers, HIF-1α and HMOX-1. (*D*) Placental expression of cytokine IFN-β. (*E*) Expression of angiogenesis markers VEGF-A, PGF, FLT-1, MMP9. (*F*) IAV polymerase (PA) mRNA expression in fetal brain. (*G* and *H*) Fetal brain mRNA expression of hypoxic markers HIF-1a and HMOX-1 (*G*) and angiogenesis markers VEGF-A and PlGF, ICAM1, and VCAM1 (*H*). (*I*) Circulating levels of sFLT in maternal plasma was measured by enzyme-linked immunosorbent assay. Quantification of circulating CFFDNA in maternal plasma was performed by ddPCR by measuring the ratio of the SRY gene (Y chromosome gene, fetal-derived) against GAPDH (maternal- and fetal-derived). Data are represented as mean ± SEM (pregnant PBS, *n* = 6 to 10; pregnant X-31, *n* = 6 to 10 of at least two or three independent experiments). All fold-change calculations of the X-31 group were measured via qPCR, performed against the PBS group within its respective timepoint, and normalized against GAPDH. Statistical analysis was performed by using an unpaired *t* test against the respective PBS control. *P < 0.05; **P < 0.01; ^#^*P* < 0.0001.

In corroboration with previous studies (15, 16), maternal IAV infection did not result in direct transplacental transmission of the virus to the fetus. At 3 dpi, we noted no IAV messenger RNA (mRNA) in the placenta of infected dams or in fetal brain (Fig. 1 *A* and *F*). Despite a lack of transplacental infection, we observed significant signs of placental dysfunction, fetal distress, and developmental complications. At both 3 and 6 dpi, there was a significant ∼10 to 20% reduction in pup and placental weights, but no alterations in the number of resorbed pups (Fig. 1*B*). In addition to placental and fetal growth restriction in the offspring of IAV-infected dams (Fig. 1*B*), we observed a hypoxic response in the placenta at 6 dpi, but not at 3 dpi (Fig. 1*C*), as evidenced by a significant increase in the expression of hypoxic-inducible factor 1α (HIF-1α) and heme oxygenase 1 (HMOX1; Fig. 1*C*). Several studies have reported the deleterious effects on placenta and fetal development of elevated IFNs during pregnancy (17, 18). Although we did not detect virus in the placentas, type I IFNs were elevated in response to maternal IAV infection. IFN-β expression was 10-fold higher in the placenta at 6 dpi, but not at 3 dpi (Fig. 1*D*). In a similar fashion to the placenta, we noted a hypoxic response to IAV infection in fetal brains at 6 dpi, but not at 3 dpi (Fig. 1*G*). This included significant increases in HIF-1α and HMOX1 (Fig. 1*G*). Based on the hypoxic gene profiles in the placenta and fetal brain, maternal IAV infection generates a systemic inflammatory phenotype that is conducive for fetal hypoxic conditions, leading to placental growth retardation, fetal IUGR, and fetal brain hypoxia.

### Maternal IAV Infection Is Associated with Placental and Fetal Brain Angiogenesis.

The maternal cardiovascular system undergoes profound changes during pregnancy to support the developing fetus (3). Abnormal adaptations or vasculature perturbations in pregnancy can lead to severe complications for both the mother and the developing fetus, including hypertension and preeclampsia. Our findings thus far indicate that maternal IAV is associated with placental and fetal brain hypoxia. Given that hypoxia is a powerful stimulus for angiogenesis, we described the effect of maternal IAV infection on the expression of the angiogenic markers VEGF-A and placental growth factor (P1GF) in the placenta and fetal brain. VEGF-A has been shown to regulate all steps of the angiogenesis process in the placenta, and inhibition of PlGF has been shown to suppress inflammation and pathological angiogenesis (19), while ameliorating maternal hypertension and preeclampsia in mice (20). Placental expression of angiogenic factors VEGF-A and PlGF, which bind to FLT1, were significantly up-regulated in pregnant dams following IAV infection at 6 dpi, but not at 3 dpi, a result consistent with the temporal aspects of hypoxia (Fig. 1*E*). In addition, we showed that placental gene expression of FLT1 was significantly increased in dams infected with IAV infection when compared to controls (Fig. 1*E*). Matrix metalloproteases (MMPs) play crucial roles in the extracellular matrix remodeling of the placenta and pregnancy complications including preterm birth and preeclampsia (21, 22). Furthermore, we observed increased placental MMP-9 expression in IAV-infected dams when compared to uninfected controls (Fig. 1*E*). We also assessed critical elements of the renin–angiotensin system, including the angiotensin AT1 and AT2 receptors, both of which were increased in the placentas of IAV-infected dams (*SI Appendix*, Fig. S2*A*). Placental hypoxia and angiogenesis can drive the release of highly inflammatory growth factors and CFFDNA. We noted elevated serum levels of sFLT1 (a marker of vascular inflammation and a protein that is elevated in preeclampsia) and cell-free levels of the SRY gene (a male-specific gene which, when elevated in maternal circulation, could be indicative of increased trophoblast apoptosis and fetal distress) in IAV-infected dams (Fig. 1*I*). Intriguingly, IAV infection was also associated with significantly elevated expression of the angiogenic markers VEGF-A and P1GF, as well as ICAM and VCAM in fetal brains at 6 dpi (Fig. 1*H*). Overall, IAV infection in pregnant dams is associated with an aberrant hypoxic and angiogenic response in the placenta and fetal brains, which may explain the reduced placental weight and fetal growth restriction evident following maternal IAV infection.

### IAV Infection during Pregnancy Resulted in a Profound Dysfunction of the Major Arteries, Including the Maternal Aorta, and Was Associated with Fetal Growth Retardation.

**Fig. 2.**
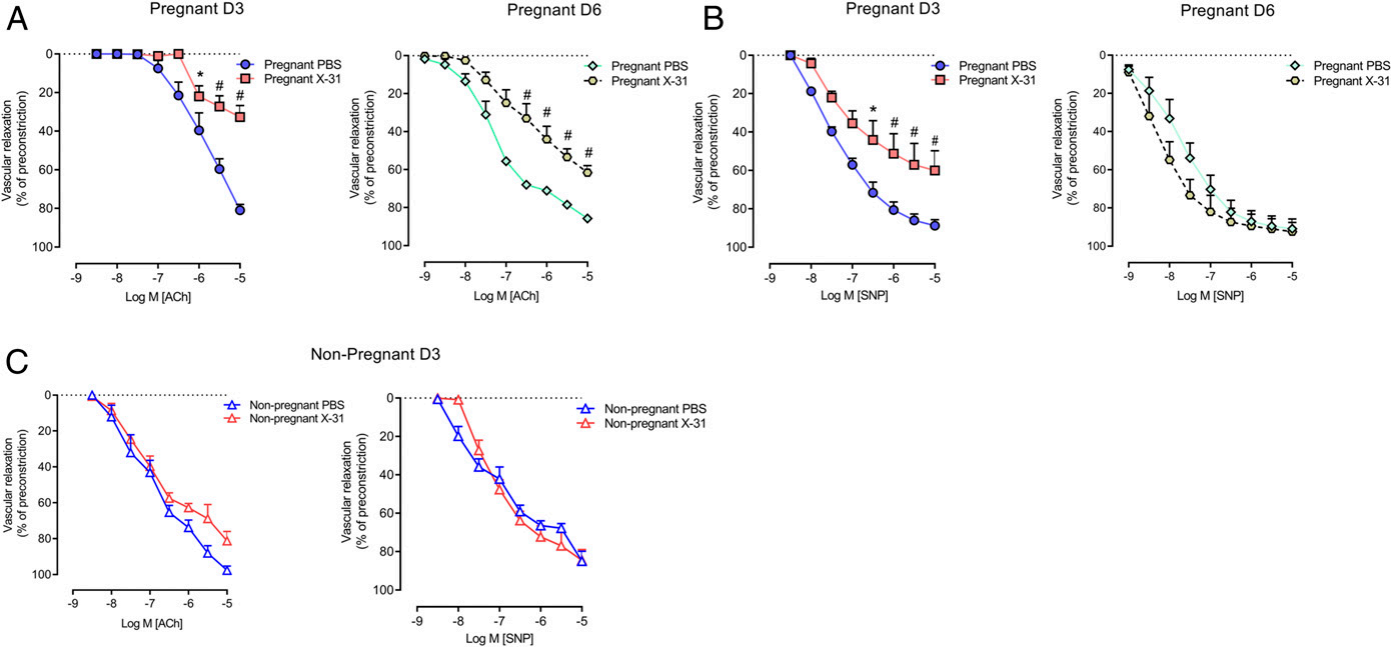
IAV infection causes vascular dysfunction in pregnant mice. Vascular reactivity was measured in isolated maternal thoracic aortic rings of pregnant and nonpregnant mice inoculated with PBS or Hk-x31 (X-31; 10^4^ PFU) for assessment at 3 dpi (D3) and 6 dpi (D6). (*A*) Endothelium-dependent vasodilation to ACh. (*B*) Endothelium-independent vasodilation to SNP. (*C*) Endothelium-dependent (ACh) and independent (SNP) vasodilation was also performed in nonpregnant mice at 3 dpi. Vascular relaxation is calculated as a percentage of preconstriction to U-46619. Data are represented as mean ± SEM (pregnant PBS, *n* = 6 to 8; pregnant X-31, *n* = 6 to 8; nonpregnant PBS, *n* = 6 of at least two independent experiments). Statistical analysis was conducted by using a two-way ANOVA followed by Holm’s Sidak post hoc multiple comparison. *P < 0.05; ^#^*P* < 0.0001.

Given that maternal IAV infection is associated with a hypoxic response in the placenta and fetal brain with a concomitant reduction in placental and fetal development, we hypothesized that IAV modifies the maternal vasculature landscape, particularly the large arteries including the aorta, and, in doing so, compromises blood flow to the placenta. To investigate vascular function, we first performed a series of functional experiments on maternal aortas using wire myography. We assessed both endothelial and smooth-muscle function using the endothelium-dependent vasodilator acetylcholine (ACh) and the endothelium-independent vasodilator sodium nitroprusside (SNP), respectively. At 3 dpi, IAV significantly impaired the ability of the thoracic aorta to relax in response to ACh with an impairment in both potency and efficacy (Fig. 2*A*). There was also a significant impairment in the relaxation response to SNP (Fig. 2*B*). Moreover, the endothelium-dependent aortic dysfunction in pregnant dams persisted until day 6 post-IAV infection (Fig. 2*A*). Strikingly, IAV infection of nonpregnant females had no effect on either ACh- or SNP-dependent relaxation in the aorta (Fig. 2*C*), strongly suggesting that this effect by IAV is pregnancy-specific. These findings demonstrated that IAV infection impairs normal vascular function in pregnancy, and, given that endothelial function was almost abolished by the infection, we speculate that this vascular phenotype will significantly impair blood flow and nutrient transfer to the placenta and fetus, contributing to placental hypoxia. The effect of IAV on vascular function is, therefore, analogous to the vascular dysfunction observed in mild and severe preeclampsia. We propose that IAV drives a preeclampsia-like syndrome during pregnancy and that this is exacerbated by IAV-induced release of hypoxic, angiogenic, and inflammatory mediators at the placental membranes.

### IAV Infection during Pregnancy Results in IAV Dissemination into the Aorta and a Proinflammatory and Pro-Oxidative Vascular Inflammatory Response.

**Fig. 3.**
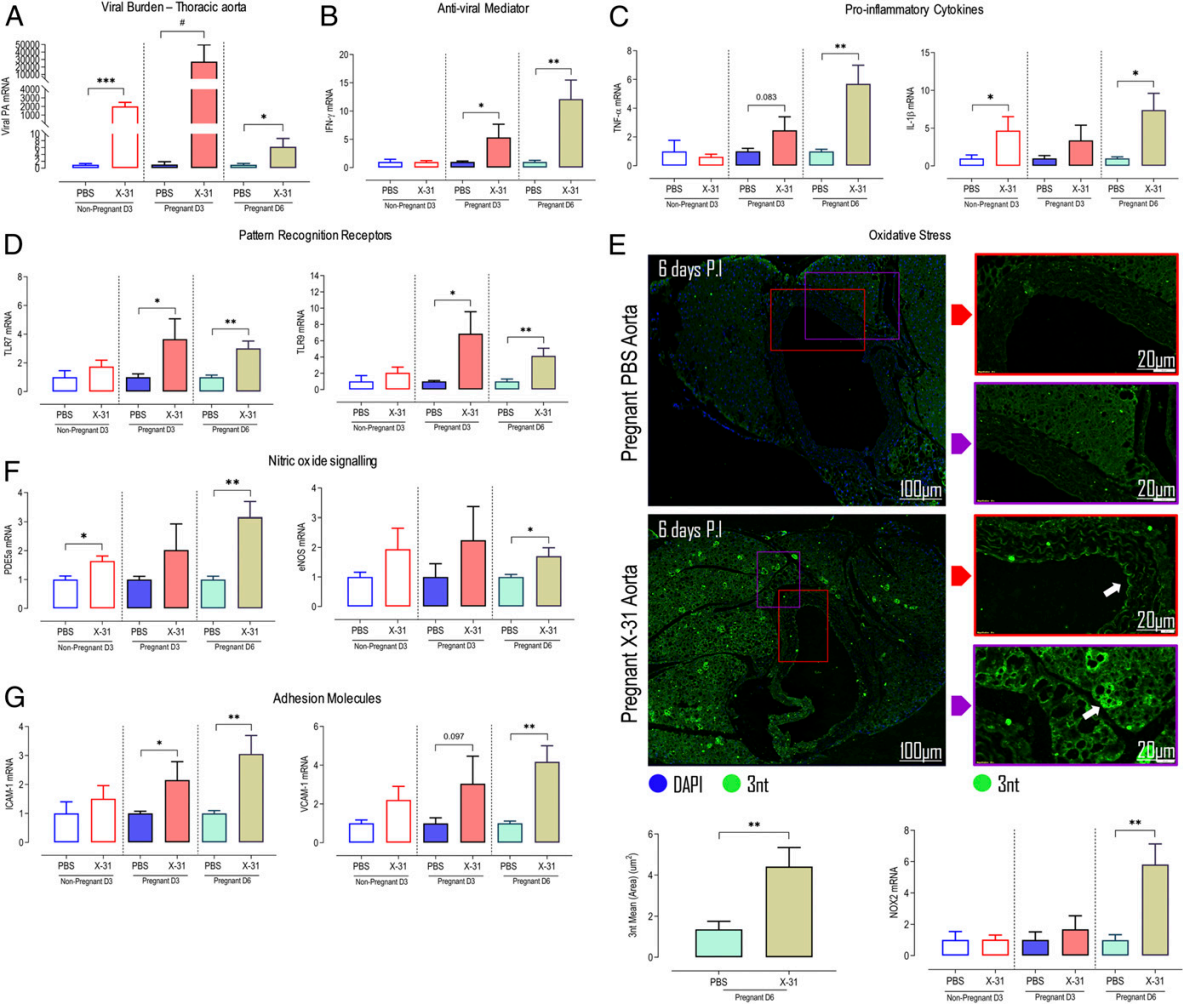
IAV disseminates into the aorta and drives a proinflammatory and oxidative stress response in the maternal vasculature. Pregnant and nonpregnant female mice were inoculated with PBS or Hk-x31 (X-31; 10^4^ PFU) for aortic assessment at 3 dpi (D3) and 6 dpi (D6). (*A*) Viral burden in maternal thoracic aorta was quantified using qPCR by measuring the IAV segment 3 polymerase (PA). (*B* and *C*) Thoracic aorta gene expression of antiviral mediator IFN-γ and proinflammatory cytokines TNF-α and IL-1β. (*D*) Aortic mRNA expression of pattern recognition receptors TLR7 and TLR9. (*E*) Immunofluorescence microscopy of pregnant PBS- and Hk-x31–infected mice labeled with 3 nitro tyrosine (3nt) antibody (green). Arrows show areas of dense peroxynitrite production. Also shown are the quantification results and oxidative stress marker NOX2 gene expression. (*F*) Endothelial NO signaling PDE5A and eNOS expression in the aorta. (*G*) Adhesion molecule ICAM1 and VCAM1 gene expression. Data are represented as mean ± SEM (pregnant PBS, *n* = 6 to 8; pregnant X-31, *n* = 6 to 8; nonpregnant PBS, *n* = 6 of at least two or three independent experiments). All fold-change calculations of the X-31 group were measured via qPCR, performed against the PBS group within its respective timepoint and normalized against GAPDH. Statistical analysis was performed by using unpaired *t* test against the respective PBS control. *P < 0.05; **P < 0.01; ***P < 0.001; ^#^*P* < 0.0001.

The immune system is known to play a central role in the pathogenesis of endothelial dysfunction in preeclampsia, as well as hypertension (23–25) and cardiovascular diseases such as atherosclerosis and myocardial infarction. Given that IAV caused impairment in vascular relaxation, we next assessed the effect of IAV on aortic inflammation. First, we detected IAV mRNA in the aorta of pregnant mice using qPCR, indicating that IAV disseminates peripherally, reaching this large blood vessel at 3 dpi (Fig. 3*A*). Interestingly, pregnancy was associated with a 10-fold higher level of viral mRNA compared to nonpregnant mice (Fig. 3*A*). Aortic viral dissemination was associated with a robust inflammatory response in the aorta, which is exacerbated during pregnancy. Specifically, we observed increased mRNA expression of antiviral mediator (IFN-γ) and proinflammatory cytokines (IL-1β and TNF-α) (Fig. 3 *B* and *C*). The pattern-recognition receptors TLR7 and TLR9 were also up-regulated in IAV-infected aortas, and these responses in the aorta persisted at 6 dpi (Fig. 3*D*). The NADPH oxidase 2 (NOX2) oxidase subunit is localized specifically to subcellular compartments called endosomes and is the primary source of inflammatory cell reactive oxygen species (ROS) during IAV infection (26–32). NOX2 expression was increased in the aortas at 6 dpi, indicative of oxidative stress (Fig. 3*E* and *SI Appendix*, Fig. S3). NOX2 oxidase-dependent superoxide anion has been shown to react with and inactivate endothelial-derived NO and, in the process, generates the highly toxic reactive nitrogen species peroxynitrite. As NO potently regulates vascular function, the formation of peroxynitrite negatively impacts NO bioavailability (33). In the pregnant influenza-infected group, oxidative stress was significantly elevated, with increased peroxynitrite deposition in the aorta, including distinct “hot pockets” of oxidative stress in the perivascular fat (Fig. 3*E*). These distinct hot pockets could possibly contain immune-cell subsets such as T cells and inflammatory macrophages and are characteristic of artery tertiary lymphoid organs (34). There was no alteration in aortic endothelial nitric oxide synthase (eNOS) mRNA expression at day 3, but an elevation was observed at 6 dpi (Fig. 3*F*). This increase in eNOS at 6 dpi could be indicative of an elevated level of uncoupled eNOS, which becomes an additional critical source of superoxide anion that contributes to vascular dysfunction (33). Moreover, there was a significant up-regulation in the expression of phosphodiesterase type 5A (PDE5A), a critical enzyme that drives cyclic guanosine monophosphate breakdown and metabolism, which will also contribute to vascular dysfunction (Fig. 3*F*). Following IAV infection, there was an increase in the expression of both angiotensin AT1 and AT2R (*SI Appendix*, Fig. S2*B*) in the aorta, which is a critical receptor for vasoconstriction in response to Ang II. We also observed a significant elevation in aortic levels of the adhesion molecules (ICAM and VCAM) (Fig. 3*G*). We assessed the same inflammatory markers in nonpregnant mice infected with IAV. In contrast to pregnant mice, IAV failed to modify the expression of these markers at day 3, and this is consistent with IAV failing to modify the function of the aorta of nonpregnant mice at this time point. It is possible that IAV might modify the vascular function in nonpregnant mice after 6 dpi. However, we anticipate that this is unlikely and probably less inflammatory for two reasons. The first is that at 3 dpi, the IAV viral load within the lungs reaches a peak, and this coincides with the peak of the host innate immune response. Therefore, any alterations induced by the host inflammatory response should have manifested at day 3 in the aorta. The second is that in nonpregnant mice, IAV had no effect on IFN-γ and CD69 expression at day 3, compared to a significant elevation in these markers in pregnant mice. These findings suggest that inflammation in the vascular wall is minimal in nonpregnant mice at the peak of the innate immune response in this mouse model and relatively less than that seen in pregnant mice. Together, these findings demonstrate that, during pregnancy, IAV infection drives a profound aortic inflammatory response, most likely when the virus disseminates into the aorta, and this has dire consequences for its function. The IAV dissemination into the aorta is conducive to driving 1) a decrease in NO bioavailability by NOX oxidase-derived oxidative stress, and 2) an increase in the vessel adhesiveness to inflammatory cells in the form of adhesion molecule up-regulation.

### IAV Infection during Pregnancy Results in a Vascular Storm Characterized by Retention of Patrolling Ly6C^low^ Monocytes and Elevated Ly6C^high^ Proinflammatory Monocytes, Neutrophils, and T Cells in the Aorta.

**Fig. 4.**
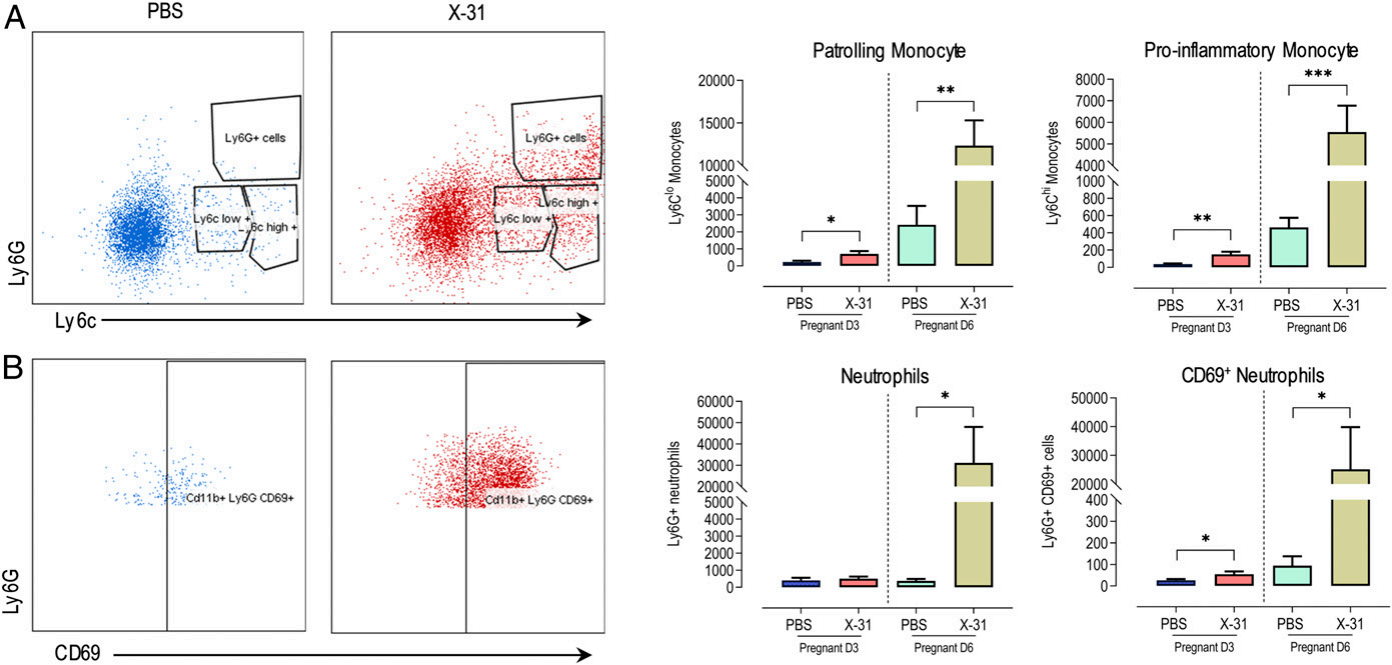
IAV infection promotes innate inflammation via the infiltration of monocytes and neutrophils in the aorta of pregnant mice. Single-cell suspensions were prepared from whole thoracic aorta digests from pregnant mice inoculated with either PBS or Hk-x31 virus (X-31; 10^4^ PFU) at 3 dpi (D3) and 6 dpi (D6) and quantified for the following cell subsets via flow cytometry. Representative dot plots and quantification are shown. (*A*) Patrolling monocytes (CD11b^+^Ly6C^low^) and proinflammatory monocytes (CD11b^+^Ly6C^high^). (*B*) Ly6G^+^ neutrophils and CD69^+^ activated neutrophils. All cell populations are measured as absolute number of CD45^+^ population per 25,000 counting beads. Data are represented as mean ± SEM (pregnant PBS, *n* = 5 or 6; pregnant X-31, *n* = 5 or 6; of at least two independent experiments). Statistical analysis was performed by using unpaired *t* test against their respective PBS control. *P < 0.05; **P < 0.01; ***P < 0.001.

We next examined whether there was an infiltration of inflammatory cells into the aorta following IAV infection. There are two subsets of monocytes that have functional effects on blood vessels, Ly6C^low^ monocytes, or “patrolling” monocytes, and Ly6C^high^ monocytes, which are proinflammatory. Ly6C^low^ monocytes are “accessory cells” of the endothelium, due to their patrolling function on the luminal side of the vessel. A critical means of regulating Ly6C^low^ monocyte function is via TLR7 activation, which is highly expressed in these cells. TLR7 is a nucleic-acid-pattern recognition receptor (PRR) that senses single-stranded RNA (ssRNA), including that from IAV. TLR7 activation increases Ly6C^low^ monocyte retention time on the endothelium and orchestrates the focal necrosis of endothelial cells, which then recruits neutrophils (35). TLR7-dependent necrosis is rapid and leaves the basal lamina, tubular epithelium, and glomerular structures intact. Therefore, Ly6C^low^ monocytes behave as “gatekeepers” of the vasculature, although it is easy to conceive that their action on endothelium during pregnancy might compromise vascular function and blood flow to the growing fetus. Thus far, we have shown that several signaling elements were detected or up-regulated at the aorta following IAV infection for the recruitment of monocytes, including; 1) IAV, 2) TLR7, and 3) ICAM up-regulation as the adhesion molecule for Ly6C^low^ monocytes (35). To examine this further, we characterized the infiltrating leukocyte subpopulations by performing a series of flow-cytometry analyses at both 3 and 6 dpi. At day 3 post-IAV infection, there was a significant elevation in patrolling CD11b^+^Ly6C^low^ monocytes, CD11b^+^Ly6C^high^ proinflammatory monocytes, and Ly6G^+^CD69^+^ activated neutrophils in the aorta (Fig. 4 *A* and *B*). This innate immune response in the aorta increased by almost 10-fold at 6 dpi with a remarkable increase in CD11b^+^Ly6C^low^ monocytes, CD11b^+^Ly6C^high^ proinflammatory monocytes, and Ly6G^+^ neutrophils (Fig. 4 *A* and *B*).

**Fig. 5.**
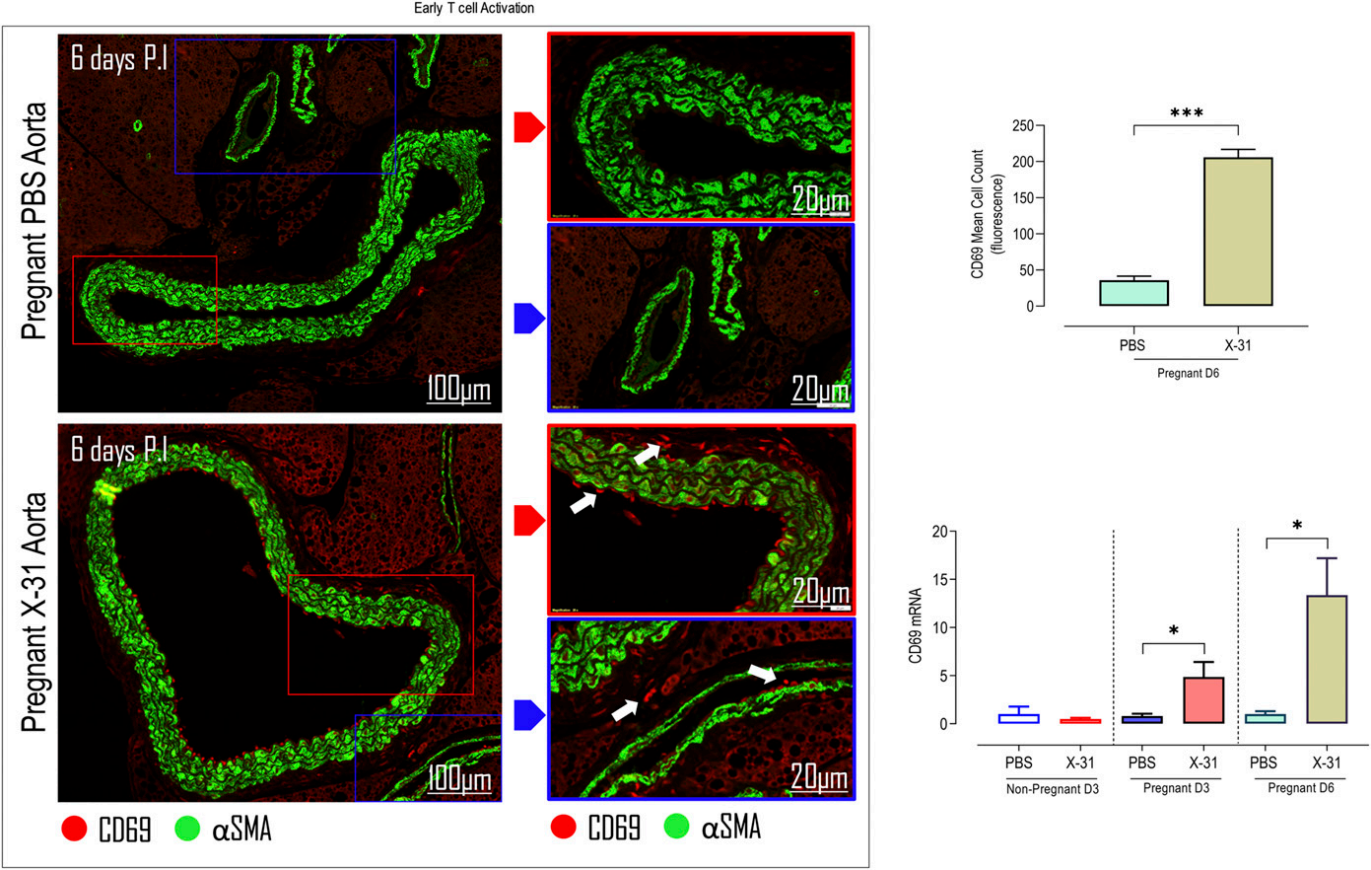
IAV infection promotes an adaptive immune T cell response in the aorta of pregnant mice. Mice were inoculated with either PBS or Hk-x31 virus (X-31; 10^4^ PFU) at 3 dpi (D3) and 6 dpi (D6). Immunofluorescence microscopy of pregnant PBS- and Hk-x31–infected mice labeled with early T cell activation marker CD69 antibody (red) are shown. Arrows show areas of positive CD69 cells and α smooth muscle actin (αSMA). Also shown are the quantification results and CD69 mRNA expression. Data are represented as mean ± SEM (pregnant PBS, *n* = 5 or 6; pregnant X-31, *n* = 5 or 6; of at least two independent experiments). Statistical analysis was performed by using unpaired *t* test against their respective PBS control. *P < 0.05; ***P < 0.001.

T cells are key contributors to vascular dysfunction in hypertension (23) via their release of IFN-γ and consequent production of ROS and impairment of NO bioavailability. ROS production by T cells is an indirect process. Evidence suggests that activated vascular T cells indirectly increase NOX1 and NOX2 oxidase subunits’ expression through the release of inflammatory mediators such as TNF-α (23, 36). Therefore, we examined whether IAV modified the numbers of circulating subpopulations of these T cells, which infiltrated the aorta at 6 dpi (*SI Appendix*, Fig. S4). Overall, we found that there were no significant alterations in circulating CD8^+^ and CD4^+^ T cells (*SI Appendix*, Fig. S4). Moreover, there were no changes in activated CD8^+^ and CD4^+^ T cells (*SI Appendix*, Fig. S4). At day 3 post-IAV infection, there were no significant alterations in T cell populations in the aorta, including CD8^+^, CD4^+^, and CD4^+^ FoxP3^+^ regulatory T cells (Tregs) or in their phenotype, i.e., CD69^+^ and CD44^+^ (Table 1 and *SI Appendix*, Fig. S7). These data suggest that only an innate immune response is occurring in the aorta at this early day-3 time point after infection. While there were no significant alterations in T cell populations in the aorta at 3 dpi, there was a substantial elevation in CD8^+^ and CD4^+^ T cells and their activation, i.e., there was enhancement in their CD69^+^ and CD44^+^ status, at 6 dpi (Table 1 and *SI Appendix*, Fig. S7). Also, we assessed the expression of T cell activation markers in IAV-infected pregnant aortas. CD69 mRNA expression (a marker of early T cell activation) in IAV-infected pregnant aortas was up-regulated during the early phase (3 dpi) and persisted during the later phases (6 dpi) of infection (Fig. 5). We confirmed CD69 expression with immunofluorescence, and an array of CD69-positive cells were detected in the aorta, particularly lining the endothelium, and within the peri-adventitial space and surrounding vaso-vasorum (Fig. 5 and *SI Appendix*, Fig. S5). These CD69 cells were located within specific pockets of the vascular walls and effectively visualized sites of pathogenesis.

**Table 1.** Quantification of aortic memory T helper (CD44^hi^ CD4^+^) and cytotoxic (CD44^hi^ CD8^+^) T cells and Treg (FOXP3^+^ CD4^+^) cells by flow cytometry between IAV-infected and control dams

	Pregnant 3 dpi			Pregnant 6 dpi		
	PBS (*n* = 7)	X-31 (*n* = 9)	*P* value	PBS (*n* = 7)	X-31 (*n* = 7)	*P* value
CD44^hi^ CD4^+^ T cells	523	403	0.353	**4,190**	**11,448**	**0.027**
CD44^hi^ CD8^+^ T cells	536	307	0.262	**2,230**	**11,775**	**0.008**
FOXP3^+^ CD4^+^ cells	2,214	2,401	0.463	3,337	6,587	0.054

Data are represented as absolute cell numbers. Statistical analysis was performed using unpaired *t* test against their respective PBS control. Boldface text indicates significance; *P* < 0.05 was considered significant.

## Discussion

These findings collectively demonstrate that IAV infection in pregnancy initiates a profound vascular inflammatory response, which we have termed a vascular storm and is defined as a disturbed state of the vasculature that alters vascular homeostasis. This is marked by a significant and excessive infiltration of activated innate and adaptive immune cells, together with the overproduction of proinflammatory cytokines IL-1β and TNF-α, adhesion molecules ICAM and VCAM, and oxidative stress mediator NOX2 within the vasculature in response to IAV. We suggest that this resulting aortic inflammation disrupts vascular homeostasis and leads to the extensive vascular dysfunction. Intriguingly, while we showed a significant level of IAV mRNA at 3 dpi in the aorta, at day 6, the level of IAV mRNA expression had almost returned to undetectable levels. Therefore, in a similar fashion to lung inflammation, we hypothesize that the aortic inflammatory response characterized here is critical for clearing IAV infection from the vasculature, but with potentially deleterious off-target effects to the developing offspring.

Taken together, we have identified major components of a severe immune overreaction to IAV in the maternal vasculature. This vascular inflammation during maternal IAV infection entails an early innate immune response, characterized by the infiltration of monocytes, macrophages, and neutrophils at blood vessels, followed by an adaptive immune response with T lymphocyte infiltration. This is associated with a substantial decrease in capacity of the blood vessels to vasodilate that is specifically necessary for blood flow toward the growing fetus. Intriguingly, the same IAV infection resulted in either no or a very mild vascular inflammatory response in nonpregnant mice. From our observations, we speculate that this robust vascular inflammation following maternal IAV infection is driven by several signaling elements, including 1) two danger signals, i.e., foreign pathogenic IAV and semiforeign CFFDNA, acting synergistically to trigger systemic inflammation; 2) elevated levels of circulating sFLT1 in maternal circulation; 3) up-regulation of aortic TLR7 (ssRNA sensor) and TLR9 (DNA sensor); 4) up-regulation of adhesion molecules for Ly6C^low^ monocyte retention to the vascular wall; 5) recruitment and activation of neutrophils and T lymphocytes; and 6) NOX2 oxidase-derived superoxide anion production in the aorta. We have previously identified that IAV promotes endosomal NOX2-derived ROS and mitochondrial ROS (mtROS) production, which can exacerbate IAV pathogenesis by driving innate immune inflammation (37). Blockade of mtROS by (2-(2,2,6,6-Tetramethylpiperidin-1-oxyl-4-ylamino)-2-oxoethyl)triphenylphosphonium chloride (mitoTEMPO) reduced innate immune-cell infiltration and their cytokine production without affecting the adaptive immune response and, thus, clearance of the virus (37). Therefore, targeting endosomal NOX2-derived ROS and mtROS production may be a potential therapeutic strategy for IAV in pregnancy by alleviating systemic and vascular inflammation.

In conclusion, we identified a hitherto-unappreciated pathology of IAV infection in pregnancy, which results in severe complications in the dams and offspring. Here, we provide evidence that IAV can disseminate into the aorta of pregnant mice to trigger a vascular storm event, which causes profound endothelial dysfunction of the major arteries, resulting in fetal distress, as characterized by placental and fetal brain hypoxia, as well as increasing circulating levels of CFFDNA and sFLT1. Our work challenges the current dogma of pregnancy-dependent immunosuppression and provides unequivocal evidence of an exacerbated immune system response to IAV in pregnancy and that this concomitantly impacts on the cardiovascular system. Our findings show a remarkable similarity in pathologies between IAV infection and preeclampsia and might therefore improve our understanding of how viral infections could trigger preeclampsia, or other hypertensive disorders, in pregnancy. Moreover, our findings in the pregnant mouse model highlight the need for specific studies in pregnant women to improve our understanding on the pathogenesis of IAV infection on the maternal cardiovascular system.

## Materials and Methods

### Animal Ethics Statement.

All animal experiments described in this manuscript were approved by the Animal Experimentation Ethics Committee of the Royal Melbourne Institute of Technology (RMIT) University Animal Ethics Committee (Ethics no. 1801). The experiments were conducted in compliance with the guidelines of the National Health and Medical Research Council of Australia on animal experimentation.

### Animals.

Eight- to 12-wk-old pregnant and age-matched nonpregnant female C57BL6/J mice were obtained from the Animal Resources Centre Western Australia and housed in the animal research facility (RMIT University) under standard conditions. Mice were placed in groups of three or four in separate cages.

### Virus.

The Hk-x31 (H3N2) mouse-adapted IAV strain was provided by Patrick Reading, Department of Immunology and Microbiology, The Peter Doherty Institute for Infection and Immunity, University of Melbourne, Melbourne. Virus aliquots were provided in phosphate-buffered saline (PBS, catalog no. D8537, Sigma) at 9.6 × 10^7^ plaque-forming units (PFU)/milliliter and stored at −80 °C until required.

### In Vivo Infection with IAV.

Pregnant mice at E12 gestation (second trimester) and age-matched-nonpregnant female mice (*n* = 6 to 8 per group) were sedated with isoflurane inhalation and inoculated intranasally (i.n.) with 35 µL of 10^4^ PFU of Hk-x31 virus diluted in PBS or mock infected with PBS only at day 0. Mice were then weighed and monitored daily.

### Airway Inflammation, Cell Differentials, and Blood Analysis.

At study endpoints, nonpregnant mice were euthanized at day 3 and pregnant mice on day 3 or 6 via an intraperitoneal injection of ketamine (180 mg/kg)/xylazine (32 mg/kg) mixture, and organs were harvested. To assess airway inflammation, the lower jaw to the top of the rib cage was incised to expose salivary glands, which were then separated to expose the smooth muscle layer on the surface of the trachea. The smooth muscle layer was removed, and a small incision was made near the top of the trachea. A sheathed 21-Gauge needle was then inserted into the lumen, and the lung was lavaged with 300 to 400 μL of PBS repeatedly and transferred to an Eppendorf tube. Cell-viability assessment involved staining total bronchioalveolar lavage fluid (BALF) cells with 10 µL of ethidium bromide solution (catalog no. 15585011, Thermofisher Scientific) and transferred to a hemocytometer to quantify the total number of viable cells.

Differential cell analysis was prepared from BALF by centrifugation of 5 × 10^4^ cells on the Cytospin 3 (Shandon) at 112 × *g* for 5 min. Slides were fixed for 1 min in propan-2-ol and air-dried in a fume hood overnight. The samples were then stained with Rapid I Aqueous Red Stain^TM^ (catalog no. RS1-1L, AMBER Scientific) for 5 min and rinsed thoroughly in water and Rapid II Blue Stain^TM^ (catalog no. RS11-1L, AMBER Scientific) for 5 min, before being washed thoroughly again in water. Slides were submerged once in 70% ethanol and twice in absolute ethanol prior to being placed twice into histolene (Grale Scientific, catalog no. 11031/5) for 5 min each. Samples were then mounted in dibutylphthalate polystyrene xylene mounting medium (catalog no. AJA3197-500ML, Labchem) and coverslipped. Analysis involved counting random fields of differentiated cells, including macrophages, neutrophils, and lymphocytes, to a total of 500 cells per sample by standard morphological criteria.

Blood was retrieved by performing a cardiac puncture to obtain between 0.3 and 1 mL of blood. For flow-cytometric analysis, 0.1 mL of clexane was pumped into the heart before blood was collected, and the amount retrieved was recorded. Blood differential analysis involved the use of the CELL-DYN emerald 22 hematology analyzer (Abbott), and, subsequently, the blood was centrifuged at 10,000 × *g* for 10 min at 4 °C to retrieve plasma to be stored at −80 °C.

### Quantification of mRNA by qPCR.

Maternal lung, thoracic aorta, three placentas, and fetal brains per dam were harvested from mice and fetus per experimental group on 3 or 6 dpi for RNA extraction using the RNeasy Mini kit (catalog no. 74104, Qiagen) as per manufacturer’s instructions. RNA sample concentration and quality were measured by using the Nanodrop 2000 Spectrophotometer (Thermo Scientific). The complementary DNA (cDNA) synthesis was performed on 1.0 to 2.0 μg of total RNA by using the High-Capacity cDNA Reverse Transcription Kit (catalog no. 4368814, Applied Biosystems). Total RNA was added to a mastermix mixture of reagents in the High-Capacity cDNA reverse-transcription kit to make a final volume of 20 μL and transcribed at the following settings: 25 °C for 10 min, 37 °C for 120 min, and 85 °C for 5 min, and kept at 4 °C until collection using the Veriti Thermal Cycler (Applied Biosystems).

qPCR was later carried out by using the TaqMan Universal PCR Master Mix (catalog no. 4304437, Applied Biosystems) or SYBR Green PCR Master Mix (catalog no. 4367659, Applied Biosystems) when measuring viral polymerase and analyzed on the Applied Biosystem QuantStudio 7 Flex Real-Time PCR System (Thermofisher). The PCR primers for TNF-α, IL-1β, IFN-β, IL-6, NOX2, CD69, CXCL2, CCL3, IFN-γ, AGRT1α, AGRT2, VCAM-1, ICAM-1, PDE5α, VEGF, HIF-1α, HMOX-1, TLR7, FLT1, MMP9, PlGF, TLR9, and eNOS were included in the Assay on-Demand Gene Expression Assay Mix (Applied Biosystems). Viral titers were measured by using oligonucleotide mouse sequence for the forward and reverse primers of the segment 3 polymerase (PA) of influenza virus. The quantitative values were obtained from the threshold cycle (Ct) number. Gene-expression analysis was performed by using the comparative Ct method. Each sample individual target gene-expression level was normalized against GAPDH mRNA expression, and the data were expressed relative to the control.

### Wire Myograph.

Maternal thoracic aortic rings were harvested and dissected free of perivascular adipose and connective tissue. Harvested vessels were placed in physiological carbogen-bubbled (95% O_2_ and 5% CO_2_) Krebs solution (composition in mmol/L: 119 NaCl, 4.7 KCl, 1.17 MgSO_4_, 25 NaHCO_3_, 1.18 KH_2_PO_4_, 5.5 glucose, and 2.5 CaCl_2_). The artery was then cut into 2-mm rings and mounted onto two stainless-steel pins on four-channel wire myograph baths (Danish myo Technology) containing Krebs. Vessels were normalized to a resting tension of 5 mN and allowed to equilibrate for 30 min before exposure to 0.5 × 10^−3^ M thromboxane A2 agonist U-46619 (catalog no. 56985-40-1, Cayman) to determine maximum smooth-muscle-dependent vasocontraction. Endothelium-dependent and -independent vasodilation were assessed by using increasing concentrations of ACh and SNP at 1 × 10^−9^ to 1 × 10^−5^ M in a submaximally contracted aorta. All experiments were conducted in duplicates and compared to mock-infected pregnant and nonpregnant controls.

### Flow-Cytometry Analysis.

Maternal thoracic and uterine arteries harvested at 3 and 6 dpi were minced by using scissors and digested in a digestion buffer (composition Collagenase type XI [catalog no. C7657-100MG, Sigma], hyaluronidase [catalog no. H3884-50MG, Sigma], and Collagenase Type I-S [catalog no. C1639-50MG, Sigma]) for 1 h with intermittent shakes to make up cell suspension. Total bone marrow cells were drained from the femur and tibia by using Hank’s balanced salt solution HBSS (catalog no. 14175095, Gibco) after hip dislocation and immersion in 70% ethanol. Cells suspensions were filtered through a 40-µm strainer, centrifuged at 400 × *g*, and washed twice with a fluorescence-activated cell-sorting (FACS) buffer. Total viable cells were then counted, resuspended in PBS, and incubated on ice for 30 min. The cells were then stained for 15 min at 4 °C with antibodies and washed twice with FACS buffer. The antibody panel used for staining, and in their different multicolor combinations, were as follows: 1:500 Alexa Fluor anti-CD45 (30-F11); 1:500 APC anti-CD3 (145-2C11); 1:1,000 PE-Cy7 anti-CD8 (53-6.7); 1:500 BV605 anti-CD4 (RM4-5); 1:500 FITC anti-Ly6C (HK1.4); 1:500 APC-Cy7 anti-Ly6G (1A8); 1:500 BV421 anti-CD11b (M1/70); 1:500 BV650 anti-CD69 (H1.2F3); 1:500 PerCP-CD44 (IM7); 1:1,000 PE anti-FoxP3 (FJK-16s), and live/dead Aqua (catalog no. L34965; Invitrogen). Following immunostaining, cells were resuspended in FACS buffer, fixed, and analyzed the following day on the FACARIA II flow cytometry with DIVA software (Becton Dickinson). Data were analyzed by using FlowJo software (Tree Star, Inc.). The cells were analyzed as a percentage of the CD45+ (live cells) and expressed in absolute numbers per 25,000 counting beads. Refer to *SI Appendix*, Fig. S6 for gating strategy.

### CFFDNA Purification and Quantification.

Circulating CFFDNA was extracted from maternal plasma by using the DNeasy Blood and Tissue Kit (catalog no. 69506, Qiagen) according to the manufacturer’s instructions. DNA concentration and quality were measured by using the Nanodrop 2000 Spectrophotometer (Thermo Scientific) and stored at −20 °C until use. A total of 10 to 60 ng of DNA was mixed with reagents in digital droplet PCR (ddPCR) Supermix for Probes (catalog no. 1863024, Bio-Rad Laboratories) and primers to quantify fetal SRY gene transcripts. Gene expression of total circulating (maternal and fetal) GAPDH levels was also quantified and used to normalized SRY gene expression. DNA was amplified at the following settings: 95 °C for 10 min, 94 °C for 30 s, 60 °C for 1 min, and 98 °C for 10 min, and kept at 4 °C until collection using the C1000 Touch thermocycler (Bio-Rad) and then quantified by using the QXDx ddPCR System (Bio-Rad).

### ELISA.

Plasma levels of sFlt1 in pregnant mice 3 and 6 dpi was quantified by using the Mouse sVEGFR1/sFLT1 DuoSet ELISA Kit (catalog no. DY471, R&D Systems) according to manufacturer’s instructions.

### Immunofluorescence Microscopy.

Maternal thoracic aorta was fixed in 10% neutral buffered formalin, embedded in paraffin, and prepared in 5-µm sections. Tissue sections underwent immunofluorescence staining protocol and were stained with primary antibody 3 nitro tyrosine (1:100) (AbCam catalog no. AB61392) to localize oxidative stress in the form of peroxynitrite and CD69 (1:100) (AbCam catalog no. AB202909) to localize positive CD69 cells. Tissues were imaged by using an Olympus S5 VS-ASW slide scanner and quantified by two separate blinded investigators via mean positive cell counts or fluorescence intensity using the Olympus cellSens Dimension Desktop Analyzer. All of the appropriate controls were performed, in that all primary and secondary antibodies combinations were used to prevent cross-reactivity.

### Statistical Analysis.

All data are expressed as the mean ± SEM. All comparisons were made within experimental groups and were performed by unpaired *t* test or one-way ANOVA followed by either a Kruskal–Wallis test, a Mann–Whitney *U* test, or a Tukey’s post hoc test when specific multiple comparisons were needed. Dose–response curve analysis for vascular reactivity studies was performed by using ANOVA for repeated measures. Statistical tests were performed by using GraphPad Prism (GraphPad Software, Version 8.2). Statistical significance was considered at *P* < 0.05.

## Supplementary Material

Supplementary File

## Data Availability

All study data are included in the article and *SI Appendix*.
