# Supplementary material for: Influenza A virus causes maternal and fetal pathology via innate and adaptive vascular inflammation in mice
Source: Proc Natl Acad Sci U S A. 2020 Sep 21;117(40):24964–73. doi: 10.1073/pnas.2006905117 (PMC7547222; doi:10.1073/pnas.2006905117)
Supplement: Supplementary File [file pnas.2006905117.sapp.pdf]

PNAS
www.pnas.org

Supplementary Information for

Influenza A virus causes maternal and foetal pathology *via* innate and adaptive vascular inflammation in mice.

Authors: Stella Liong[1†*], Osezua Oseghale[1†], Eunice E. To[1], Kurt Brassington[1], Jonathan R. Erlich[1], Raymond Luong[2], Felicia Liong[1], Robert Brooks[3], Cara Martin[4-7], Sharon O'Toole[4-7], Antony Vinh[8], Luke A.J. O'Neill[9], Steven Bozinovski[1], Ross Vlahos[1], Paris C Papagianis[1], John J. O'Leary[4-7], Doug A. Brooks[3,4] and Stavros Selemidis[1*]

*Correspondence to: Stella Liong (S.L); Stavros Selemidis (S.S)
Email: stella.liong@rmit.edu.au (S.L); stavros.selemidis@rmit.edu.au (S.S)

This PDF file includes:

Figures S1 to S7

www.pnas.org/cgi/doi/10.1073/pnas.2006905117

Supplementary Figures

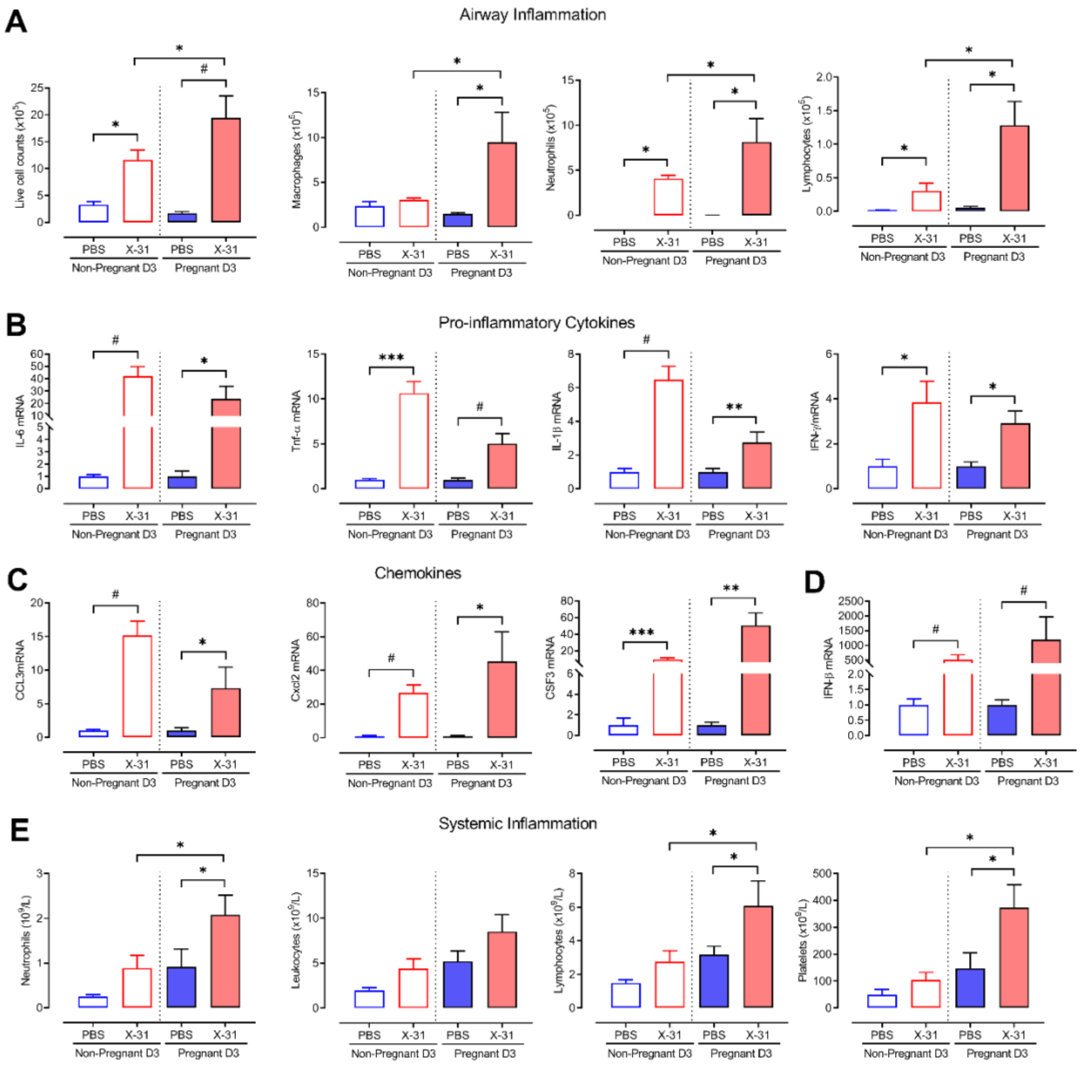

Figure S1. Influenza A virus infection in pregnancy results in an exacerbated local and systemic inflammatory response compared to non-pregnant mice. Local and systemic pro-inflammatory profiles against Hk-x31 IAV infection (X-31; 10_4 PFU) in pregnant and non-pregnant female mice were characterised at Day 3 and 6 post infection. (A) Airway inflammation was assessed by counting the number of total live bronchoalveolar lavage fluid cells from pregnant IAV infected mice. The number of infiltrating macrophages, neutrophils and lymphocytes was measured by standard morphological criteria from 500 cells in random fields (B) Gene expression analysis of inflammatory cytokines TNF-α, IL-6, IL-1β and IFN-β in the lung was performed *via* qPCR and normalized against GAPDH. (C - D) Lung mRNA expression of chemokines CCL3, CXCL2 and G-CSF; and anti-viral mediator IFN-γ was normalized against GAPDH. All fold change calculations were performed against the PBS group within its respective timepoint. (E) Maternal systemic

inflammation, including leukocytes, lymphocytes, neutrophils, platelets and mean platelet volume was measured from whole blood using a hematology analyzer. Data are represented as mean ± SEM (pregnant PBS, n=9-10; pregnant X-31, n=6; non-pregnant PBS n=6 of at least two independent experiments). Statistical analysis was performed using unpaired t-test. * P<0.05 **P<0.01, ***P<0.001.

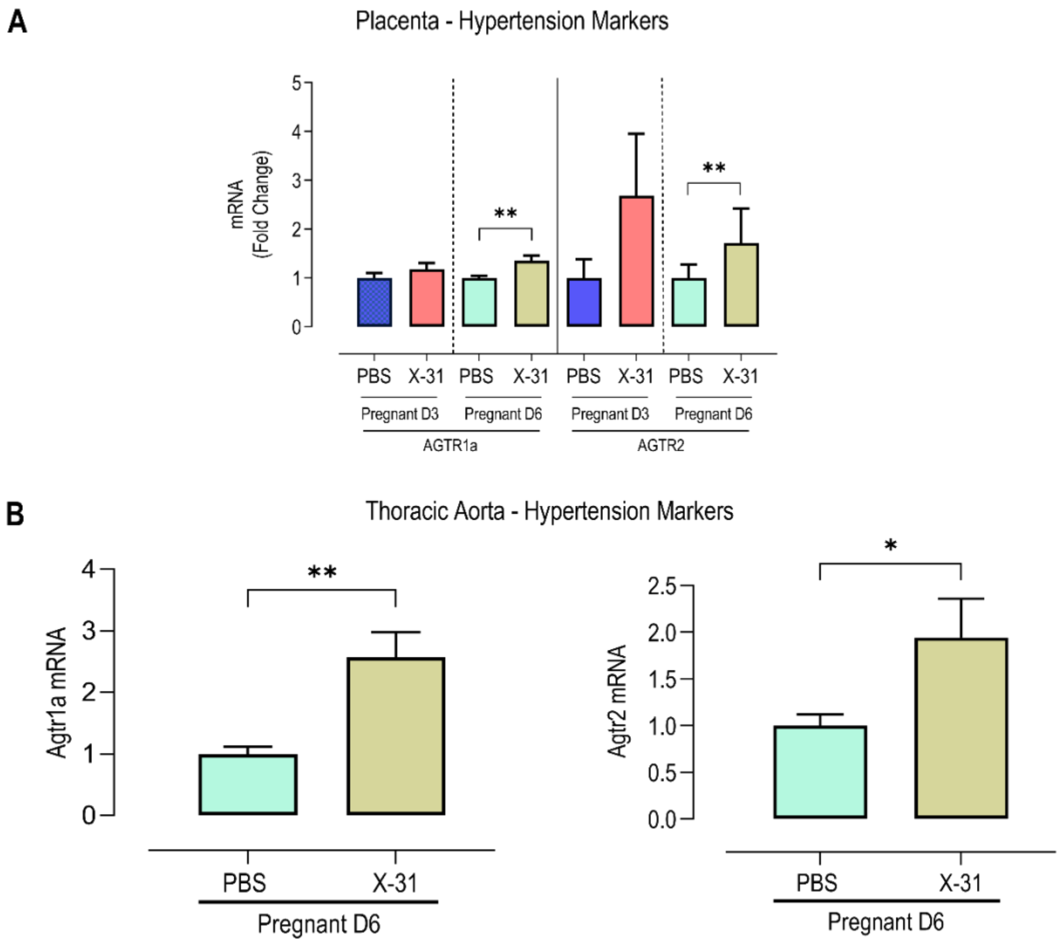

Figure S2. IAV infection induces a hypertensive phenotype in thoracic aorta and placenta in pregnant mice. Pregnant and non-pregnant female mice were inoculated with PBS or Hk-x31 (X-31; 10^4 PFU) for aortic assessment at Day 3 and 6 post infection. (A) Hypertension markers AGTR1a and AGTR2 gene expression in pregnant placenta. Hypertension markers AGTR1a and AGTR2 gene expression in pregnant aorta at day 6 p.i. (B) Data are represented as mean ± SEM (pregnant PBS, n=6-8; pregnant X-31, n=6-8; non-pregnant PBS n=6 of at least two to three independent experiments). All fold change calculations of the X-31 group were measured via qPCR, performed against the PBS group within its respective timepoint and normalised against GAPDH. Statistical analysis was performed using unpaired t-test against the respective PBS control. * *P*<0.05 and ***P*<0.01.

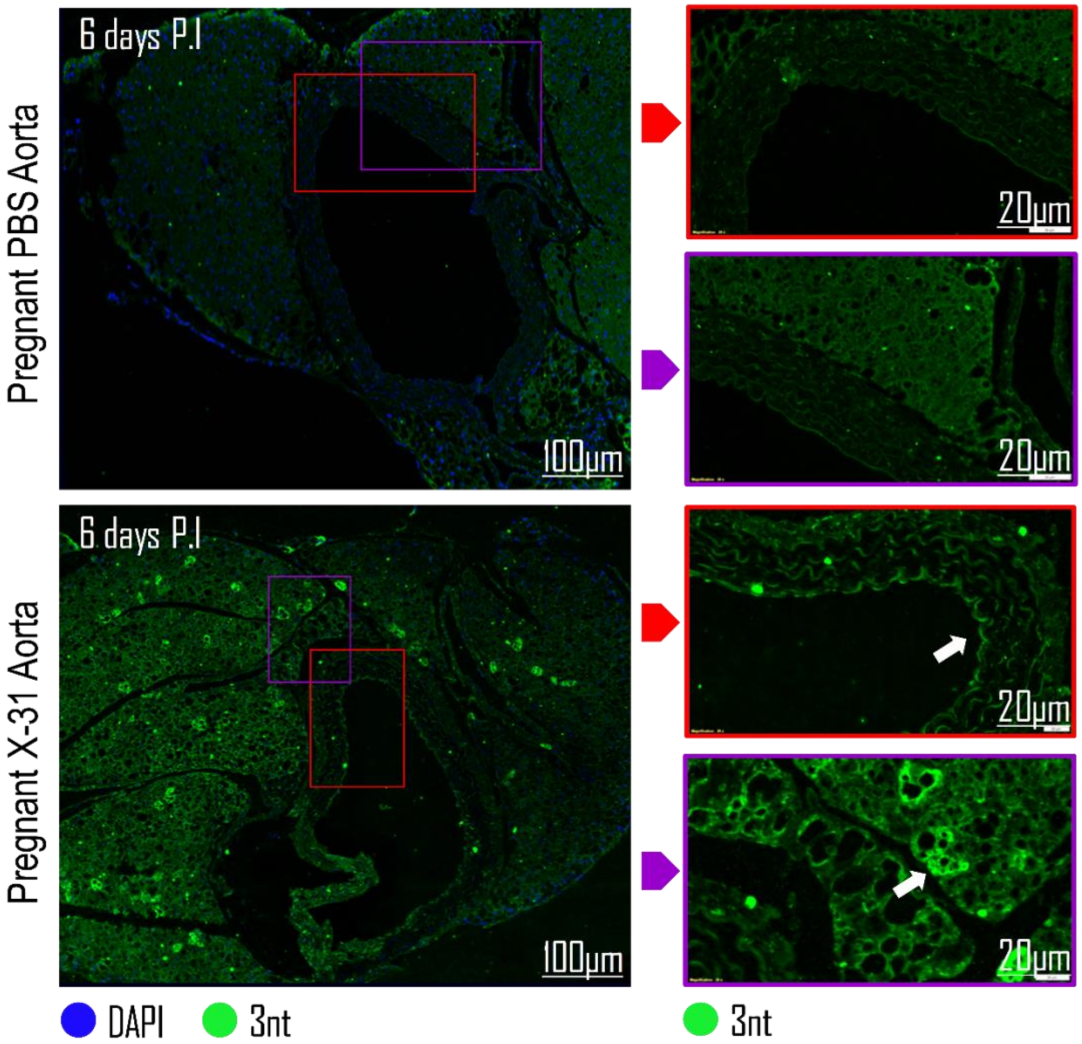

Figure S3. Representative immunofluorescence microscopy image of 3 nitro-tyrosine staining in thoracic aorta from pregnant uninfected and infected mice. Note, this supplementary Figure is identical Figure 3E and is shown here for clarity purposes.

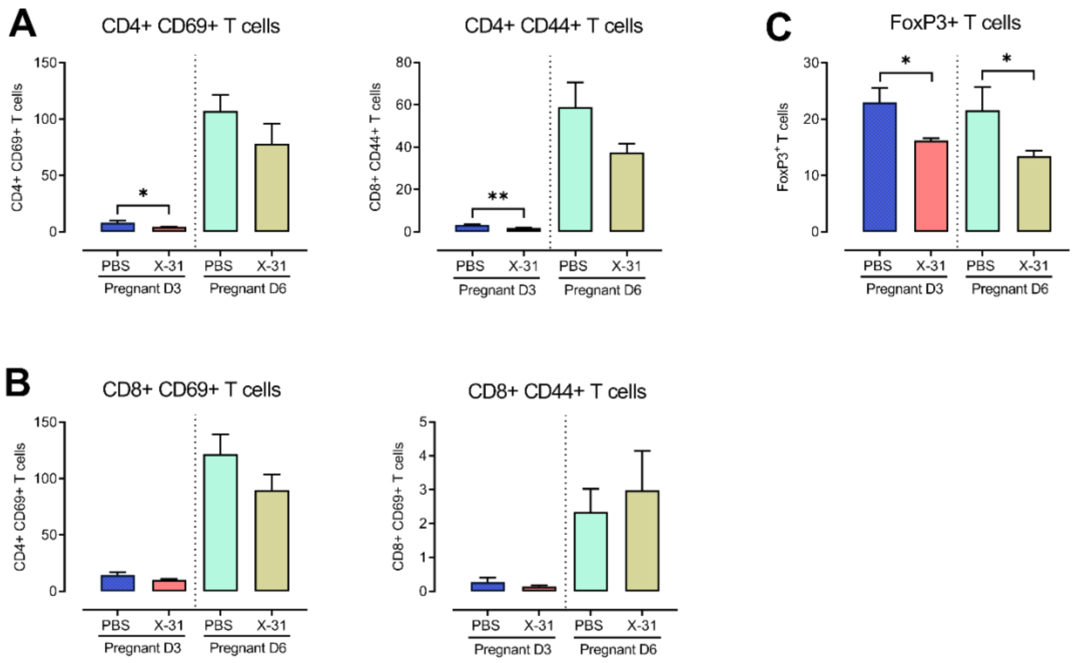

Figure S4. Systemic T cells following IAV infection during pregnancy. Single cell suspensions were prepared from whole blood and the following immune cell subtypes were quantified via flow cytometry. (A) $CD69^+$ and $CD44^+$ activated $CD4^+$ T cells. (B-C) $CD69^+$ and $CD44^+$ activated $CD8^+$ T cells and $FOXP3^+$ T cells. All cell populations are measured as absolute number of $CD45^+$ population per 25,000 counting beads. Data are represented as mean ± SEM (pregnant PBS, n=9-10; pregnant X-31, n=6; nonpregnant PBS n=6 of at least two independent experiments). Statistical analysis was performed using unpaired t-test. * P<0.05; **P<0.01.

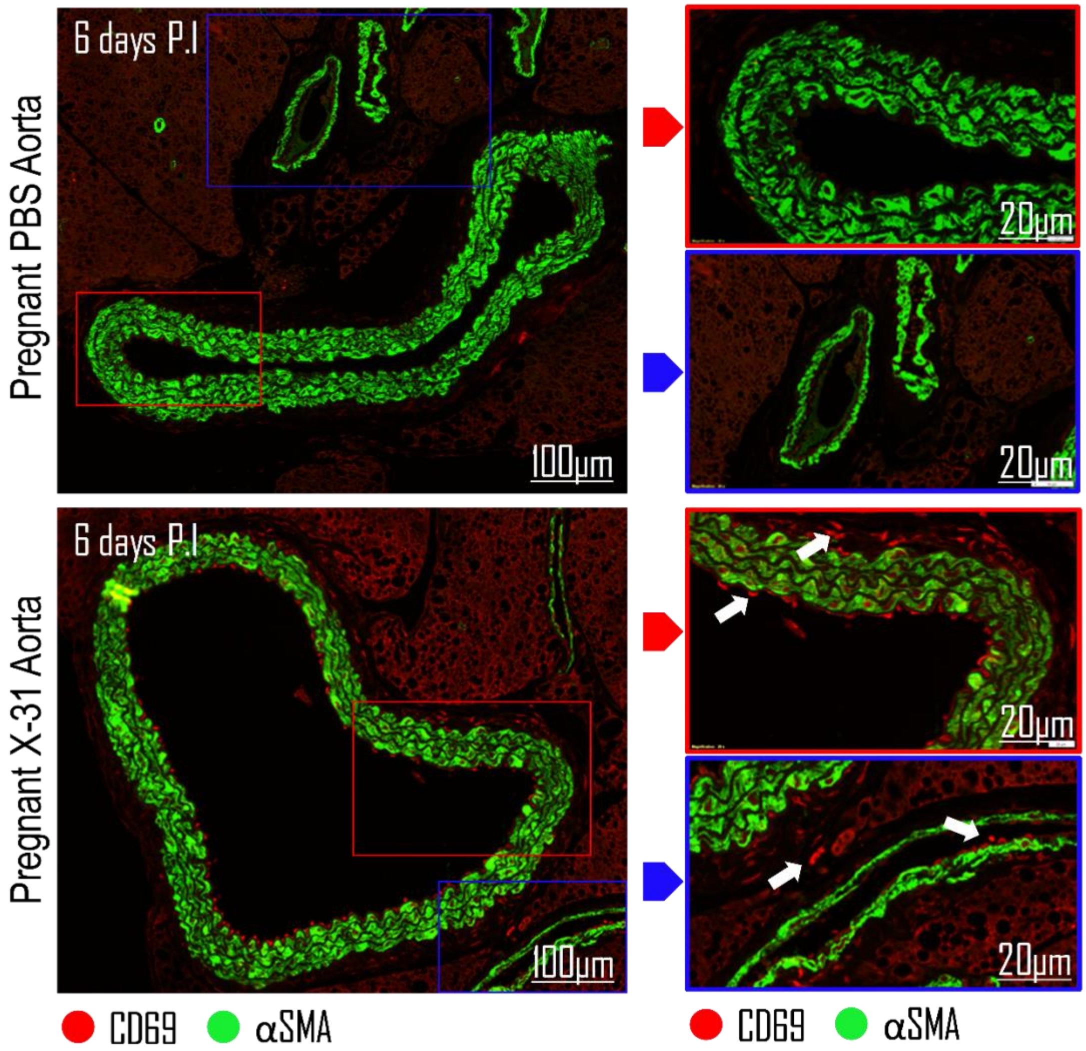

Figure S5. Representative immunofluorescence microscopy image of CD69 staining in thoracic aorta from pregnant uninfected and infected mice. Note, this supplementary Figure is identical Figure 5D and is shown here for clarity purposes.

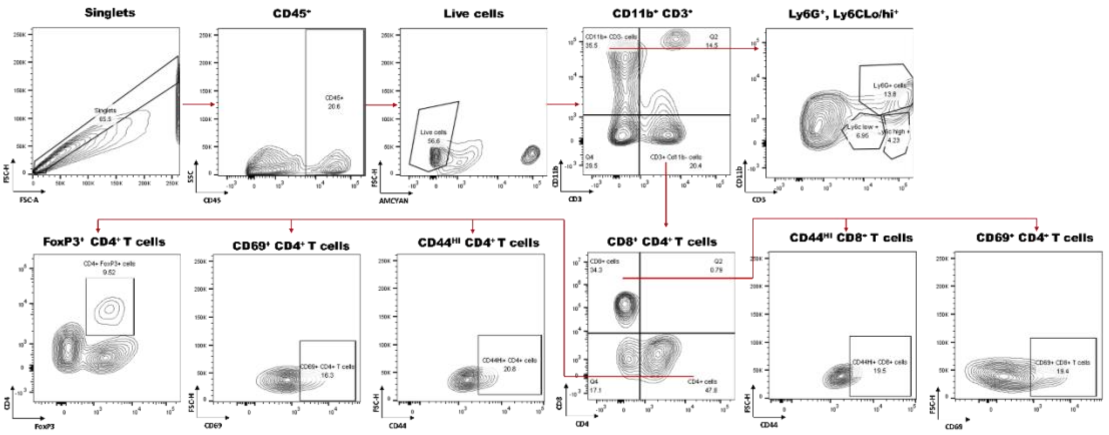

Figure S6. Gating strategy for maternal thoracic aorta single cell suspension. Macrophages and T cells were gated as $CD11b^{+}$ and $CD3^{+}$ respectively from $CD45^{+}$ Lymphocytes. Neutrophils ($Ly6G^{+}$), patrolling monocytes ($Ly6C_{low}$) and pro-inflammatory monocytes ($Ly6C_{high}$) were identified within $CD11b^{+}$ macrophages. $CD3^{+}$ T cells were further divided into subsets cytotoxic ($CD8^{+}$) and T helper ($CD4^{+}$) T cells. T regulatory cells ($FoxP3^{+}$) was identified within $CD4^{+}$ T cells subset. T cell activation and early T cell activation markers $CD44^{+}$ and $CD69^{+}$ respectively were identified within $CD4^{+}$ and $CD8^{+}$ T cell subsets.

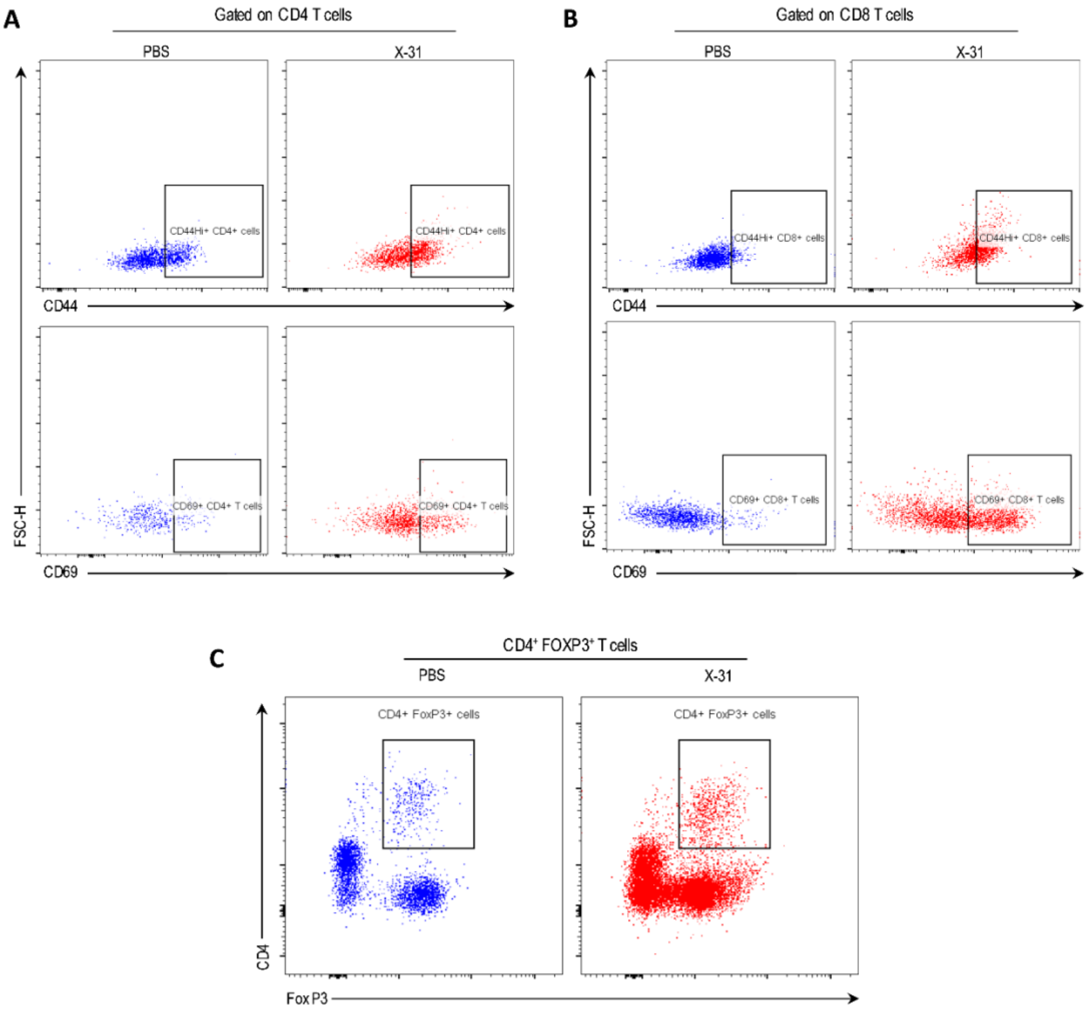

Figure S7. Representative FACS plots of activated CD4$^+$, CD8$^+$ and FOXP3$^+$ T cells in the aorta in response to influenza A virus infection in pregnant mice. Single cell suspensions were prepared from whole thoracic aorta digests from pregnant. Mice inoculated with either PBS or Hk-x31 virus (X-31; 10^4 PFU) at Day 3 and 6 post infection and quantified for the following cell subsets *via* flow cytometry. Representative dot plots showing (A) CD44^{hi}, CD69$^+$ activated CD4$^+$ and CD8$^+$ T cells. (B) CD44^{hi} and CD69$^+$ activated CD8$^+$ T cells (C) FOXP3$^+$ activated CD4$^+$ T cells.
